# Divergent dislocation of the carpometacarpal joints: a case report

**DOI:** 10.1186/s13256-018-1695-y

**Published:** 2018-06-07

**Authors:** Redouane Hani, Idriss Jeddi, Mohamed Saleh Berrada

**Affiliations:** 1 Orthopedic Surgery Department of Ibn Sina Hospital, University Mohamed V, Rabat, Morocco; 2 Foyer des Médecins internes, Souissi, Rabat, Morocco

**Keywords:** Dislocation, Fracture, Carpometacarpal joints, Reduction

## Abstract

**Background:**

Divergent carpometacarpal joint dislocations of the fingers are very rare. Due to severe swelling and overlapping of bones on a radiograph of the wrist and hand, dislocations are missed. The purpose of this clinical case report is to highlight this unusual injury to avoid missing diagnosis.

**Case presentation:**

We report a case of a 24-year-old Moroccan man, an athlete, who presented divergent carpometacarpal joint fracture-dislocations of the ulnar four fingers after a fall during a national cycling competition. Radiographs showed divergent dislocation and associated fractures. He underwent open reduction and fixation with percutaneous Kirschner wires followed by 6 weeks of immobilization. Active physiotherapy was started and the results were satisfactory after a 2-year follow-up.

**Conclusions:**

Divergent carpometacarpal joint dislocations of the fingers are exceptional; their diagnosis is sometimes difficult and may go unnoticed especially in a patient with polytrauma. The functional prognosis depends on the precocity of diagnosis and the quality of the reduction and rehabilitation.

## Background

Carpometacarpal (CMC) joint dislocations of the fingers are uncommon injuries [1]. These injuries mainly occur in young adults and represent less than 1% of all lesions of the hand [2]. Simultaneous CMC dislocations may be dorsal and volar. Dorsal dislocations are more frequent [3]. The reason why dorsal dislocations are commoner is that stronger static (dorsal ligaments) and dynamic (wrist extensors) restraints may cause the failure of bone dorsally, with the subsequent rupture of the volar ligaments [4]. The increased mobility on the ulnar side may predispose to the known greater frequency of the injury. Stability at the finger CMC joints is provided by a system of four ligaments, namely the dorsal metacarpal, the palmar metacarpal, and the two sets of interosseous ligaments. Divergent varieties are exceptional being the result of a compound traumatic mechanism [5]. The diagnosis of this unusual form of injury requires a high index of suspicion, vigilant examination, and high quality radiography. CMC joint fracture dislocation can be treated by closed reduction immobilization, closed reduction internal fixation, or open reduction internal fixation with Kirschner (K) wires. However, in cases of closed reduction, there is a higher risk of radiolocation of the CMC joint, as compared to open reduction. Open reduction and internal fixation is the recommended treatment for CMC joint dislocation [6]. Due to severe swelling and overlapping of bones on radiographs of wrist and hand, dislocations are missed.

We report the case of a 24-year-old man who presented a CMC fracture-dislocation of the four last fingers of his right hand following a fall in a national cycling competition who had received emergency treatment.

## Case presentation

**Fig. 1:**
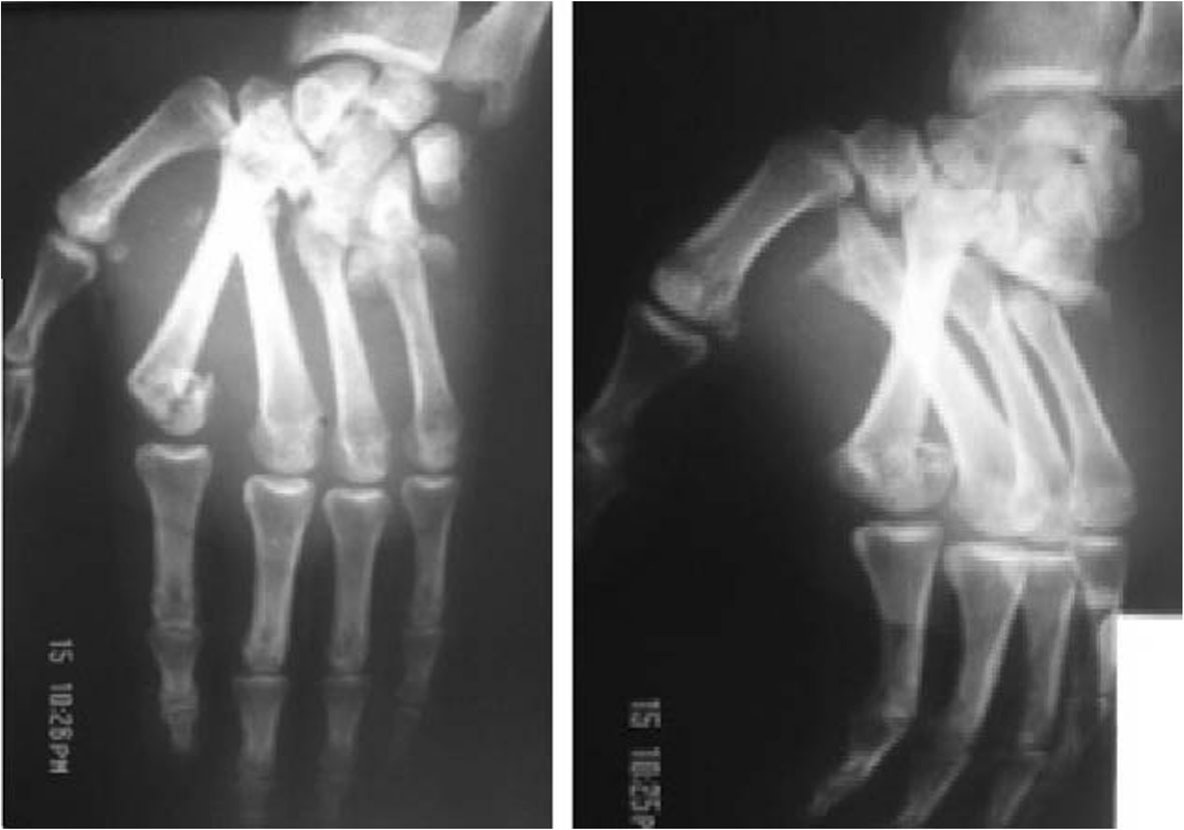
Divergent carpometacarpal dislocation of the last four fingers with fracture of the head of the second metacarpal and the fifth metacarpal base

**Fig. 2:**
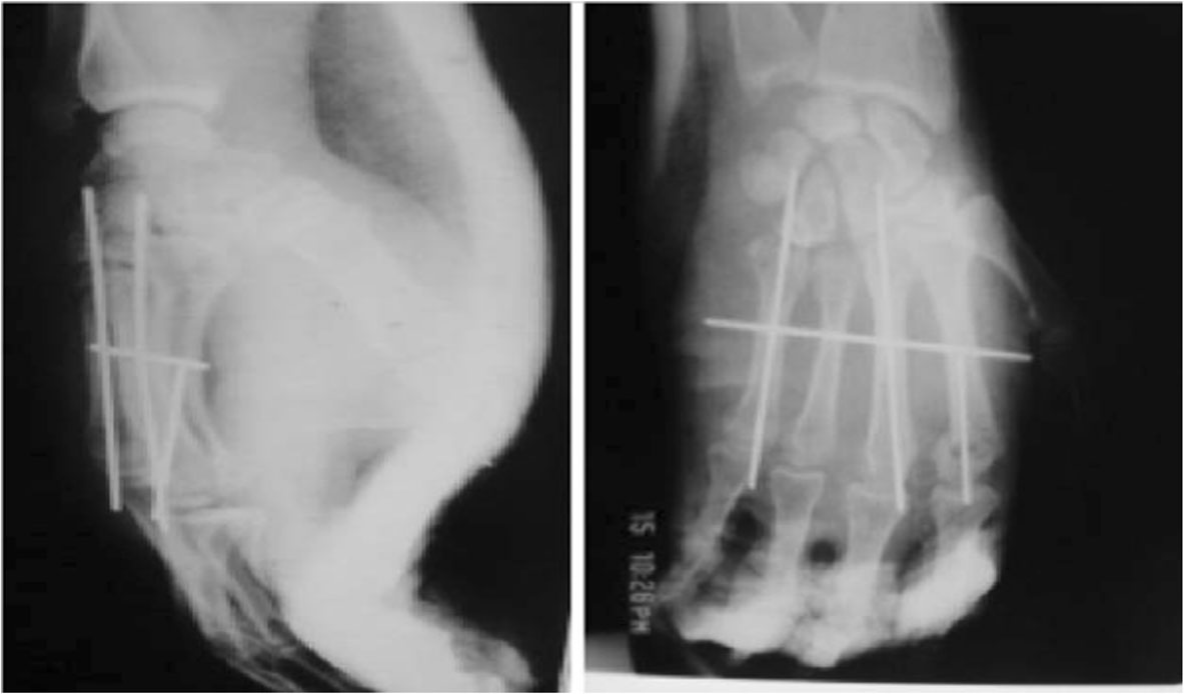
Postoperative radiograph after open reduction and stabilization by an axial pinning of the second, third, and fifth carpometacarpal joints under fluoroscopic control

A 24-year-old Moroccan man, an athlete, was admitted to the emergency department of the Ibn Sina University Hospital with complaints of relentless pain, discomfort, and inability to move his dominant right hand following a fall onto his right upper limb during a national cycling competition. He had no pathological antecedents and did not present any history of past surgery or trauma. He was unable to recall the exact mechanism of the injury. An initial clinical examination found that he was hemodynamically stable. An examination of his right upper limb showed a significant edema and a clear deformation at the dorsal face of his right hand with no signs of nerve compression. A vascular examination was normal. Radiographs of his hand showed a divergent CMC fracture-dislocation of the last four fingers, as well as associated fractures of the head of the second and the base of the fifth metacarpals. The last three metacarpals had palmar displacement while the second metacarpal was dislocated posteriorly (Fig. 1). He was immediately admitted to our operating room and underwent open reduction with dorsal approach under aseptic precautions. Two longitudinal incisions were made in the second and fourth web space addressing adjacent respective joints. CMC joint and fractures were exposed and reduction was visually achieved using external maneuvers (traction in the axis of each finger with pressure on the base of the luxated metacarpal) followed by an axial pinning of the second, third, and fifth CMC joints under fluoroscopic control (Fig. 2). Additional plastered immobilization by an intrinsic plus splint was applied for 6 weeks. Our patient underwent physiotherapy and assisted active exercises to increase strength of grip. Pins removal was done at the eighth week; approximately 10 weeks after removal of the cast, he returned to work. Clinical and radiologic examination at the 24-month follow-up visit showed no recurrence of the dislocation or arthritic phenomena. Grip strength and wrist mobility recovered, and there was no pain.

## Discussion

Dislocations and fracture-dislocations of the CMC joints are uncommon, although not to the degree implied by the scant amount of literature on the subject. Dislocation of all four medial metacarpals is rare. These injuries mainly occur in young adults. Road traffic accidents and violent trauma are the main etiology [7].

Clinical diagnosis is sometimes difficult due to the edema that takes place early and masks the deformity. This injury may be missed in an acute setting in a busy accident and emergency unit. Swelling around the wrist with shortening of the knuckle should alert the clinician toward the possibility of such an injury. In these cases, radiology remains an important diagnostic benefit although interpretation of radiographic images is sometimes difficult [8]. On routine anteroposterior view, evaluation of CMC joint is done by parallel “M lines” as described by Gilula [9]; overlap of joint surfaces, loss of parallelism, and asymmetry at the CMC joints should raise suspicion of the possibility of a subtle CMC injury. This article highlights the importance of a high index of suspicion, a true lateral radiograph, and careful evaluation of radiographs in diagnosing these injuries. Some authors recommend a computed tomography scan for a better analysis of the lesions and to detect any associated lesions unnoticed by standard radiographs. CMC dislocations are classified into: complete or partial spatula dislocations; palmar dislocations; lateral dislocations, particularly of the fifth ray; and divergent dislocations [3–9].

CMC joint fracture dislocation can be treated by closed reduction immobilization, closed reduction internal fixation, or open reduction internal fixation with K-wires. However, in cases of closed reduction, there is a higher risk of redislocation of CMC joint, as compared to open reduction. Many authors recommend open reduction especially in fracture dislocations to guarantee anatomical reduction [1, 4, 7, 9]. Orthopedic reduction is usually possible when the dislocation is recent and of less than 10 days. Hartwig and Louis considered that it is not always necessary to stabilize all the rays; for them, the stabilization of the second and third rays is the key to reduction due to the integrity of the intermetacarpal ligaments [10]. Physiotherapy of hand and wrist joint is required after 6 weeks of immobilization to avoid postoperative stiffness [11]. Several complications have been reported in the literature, such as persistence of residual hand pain, decrease of the gripping force, subluxations, and secondary displacements [12]. In addition, Lawlis and Gunther reported that patients with dislocation of the four CMC joints have better results than those with dislocation of the second and third rays [1].

## Conclusions

Divergent CMC joint dislocations of the fingers are exceptional; their diagnosis is sometimes difficult and may go unnoticed especially in a patient with polytrauma. The functional prognosis depends on the precocity of diagnosis and the quality of the reduction and rehabilitation.

## Abbreviation

CMC: Carpometacarpal
K: Kirschner
